# Preparatory activity of anterior insula predicts conflict errors: integrating convolutional neural networks and neural mass models

**DOI:** 10.1038/s41598-024-67034-5

**Published:** 2024-07-19

**Authors:** Neda Kaboodvand, Hanie Karimi, Behzad Iravani

**Affiliations:** 1 Department of Clinical Neuroscience, Karolinska Institute, Stockholm, Sweden; 2 Department of Neurosurgery, Stanford University, Stanford, CA USA; 3 School of Medicine, Tehran University of Medical Sciences, Tehran, Iran; 4 Department of Neurology and Neurological Sciences, Stanford University, Stanford, CA USA

**Keywords:** Cognitive control, Insula, Deep learning, Neurostimulation, Intrinsic neuromodulation, Neuroscience, Cognitive neuroscience, Computational neuroscience

## Abstract

Preparatory brain activity is a cornerstone of proactive cognitive control, a top-down process optimizing attention, perception, and inhibition, fostering cognitive flexibility and adaptive attention control in the human brain. In this study, we proposed a neuroimaging-informed convolutional neural network model to predict cognitive control performance from the baseline pre-stimulus preparatory electrophysiological activity of core cognitive control regions. Particularly, combined with perturbation-based occlusion sensitivity analysis, we pinpointed regions with the most predictive preparatory activity for proactive cognitive control. We found that preparatory arrhythmic broadband neural dynamics in the right anterior insula, right precentral gyrus, and the right opercular part of inferior frontal gyrus (posterior ventrolateral prefrontal cortex), are highly predictive of prospective cognitive control performance.  The pre-stimulus preparatory activity in these regions corresponds to readiness for conflict detection, inhibitory control, and overall elaborate attentional processing. We integrated the convolutional neural network with biologically inspired Jansen-Rit neural mass model to investigate neurostimulation effects on cognitive control. High-frequency stimulation (130 Hz) of the left anterior insula provides significant cognitive enhancement, especially in reducing conflict errors, despite the right anterior insula’s higher predictive value for prospective cognitive control performance. Thus, effective neurostimulation targets may differ from regions showing biomarker activity. Finally, we validated our theoretical finding by evaluating intrinsic neuromodulation through neurofeedback-guided volitional control in an independent dataset. We found that left anterior insula was intrinsically modulated in real-time by volitional control of emotional valence, but not arousal. Our findings further highlight central role of anterior insula in orchestrating proactive cognitive control processes, positioning it at the top of hierarchy for cognitive control.

## Introduction

Proactive cognitive control is a vital aspect of human brain function, enabling us to optimize attention, perception, and inhibition, ultimately enhancing cognitive flexibility and adaptive attention management. It relies on preparatory brain activity, with its exact mechanisms not yet fully understood due to limitations in neuroimaging and analytical methods^1^. Distinct neurocognitive networks manage adaptive task control^2^ and those responsible for proactive control are different from networks responsible for reactive control^1,3^. These networks may also operate at different time scales.

Functional neuroimaging has been instrumental in revealing specific frontal structures tied to diverse cognitive tasks, such as the posterior inferior frontal cortex and frontal operculum/anterior insula^4^. It has also provided evidence suggesting that as the prefrontal cortex matures, it helps enhance efficient preparatory mechanisms, reducing real-time processing demands, and facilitating the transition from reactive (sensorimotor dominance) to proactive cognitive control^5–7^. The frontal operculum/anterior insula cortex plays a central role in goal-directed cognitive control, orchestrating different functional systems involved in affect and sensory-motor processing, as well as cognitive^8,9^ and motivational processes^10,11^.

Nevertheless, the overrepresentation of functional magnetic resonance imaging (fMRI) in the literature, given its low temporal resolution, limits our quantitative understanding of the preparatory brain dynamics and impedes the applicability of any derived biomarker for closed-loop neuromodulation. High temporal resolution data in the order of milliseconds, such as electroencephalography (EEG), is essential for assessing these dynamics. Post-stimulus event-related EEG analyses indicated the early involvement and lateralized function of the anterior insula in perceptual decision-making^12^. However, most EEG studies primarily rely on analysis of grand average baseline-normalized responses, often overlooking pre-stimulus preparatory activities, limiting their ability to capture the temporal dynamics of proactive cognitive control^13^. Rare but significant efforts have examined pre-stimulus event-related potentials (ERPs) during a cognitive control task, revealing the pivotal role of anticipatory brain activity in frontal structures like the inferior frontal gyrus and anterior insula for proactive cognitive control^1,14^. However, pre-stimulus ERPs are distinct from preparatory electrophysiological activity (PEA) which reflects the brain's baseline state without anticipation.

Overreliance on univariate assessment of the grand average EEG waveforms has limited EEG's capacity to comprehend the unexplored relationship between PEA and cognitive control, particularly at finer sub-trial brain activity levels. Multivariate data-driven analysis is required for assessing the dynamic composition of the ongoing brain activity across different regions, each with unique cognitive control traits. Our recent work demonstrates the effectiveness of convolutional neural networks (CNNs) in robustly detecting relevant neural activity patterns for optimal brain function^13^. Yet, given that observed statistical associations between neural activity and behavior offer limited insights into mechanisms, a combination of counterfactual conditions and neurostimulation is required to establish causality^15^.

With the primary goal of elucidating causal mechanisms underlying proactive cognitive control and to inform the design of future neurostimulation interventions, we used two open-access datasets: 1) concurrent EEG-fMRI data during conflict-inducing cognitive control tasks (namely Stroop and Simon tasks)^16^, and 2) concurrent psychophysiological and fMRI (psychophys-fMRI) recording during real-time neurofeedback-guided volitional control^17,18^. We developed a novel multivariate, data-driven approach to comprehensively examine the preparatory EEG activity in cognitive control. Specifically, we proposed a neuroimaging-informed CNN model to predict prospective cognitive control performance from PEA, along with perturbation-based occlusion sensitivity analysis, to uncover brain regions with the most predictive PEA for proactive cognitive control. Subsequently, we adapted Jansen-Rit neural mass modeling, to integrate functional neuroimaging and electrophysiological data^19,20^, allowing us to simulate the effect of different neurostimulation protocols on PEA^21^. The simulated PEA was fed into the trained CNN model to compare the effect of different neurostimulation protocols on proactive cognitive control. To further validate our model-based findings, we used the experimental neuromodulation data^20,21^, where real-time measurement of electrophysiological-driven activity was used as a source of feedback to guide volitional control (i.e., intrinsic neuromodulation) of affective processing.

## Results

### Behavioral data

**Figure 1:**
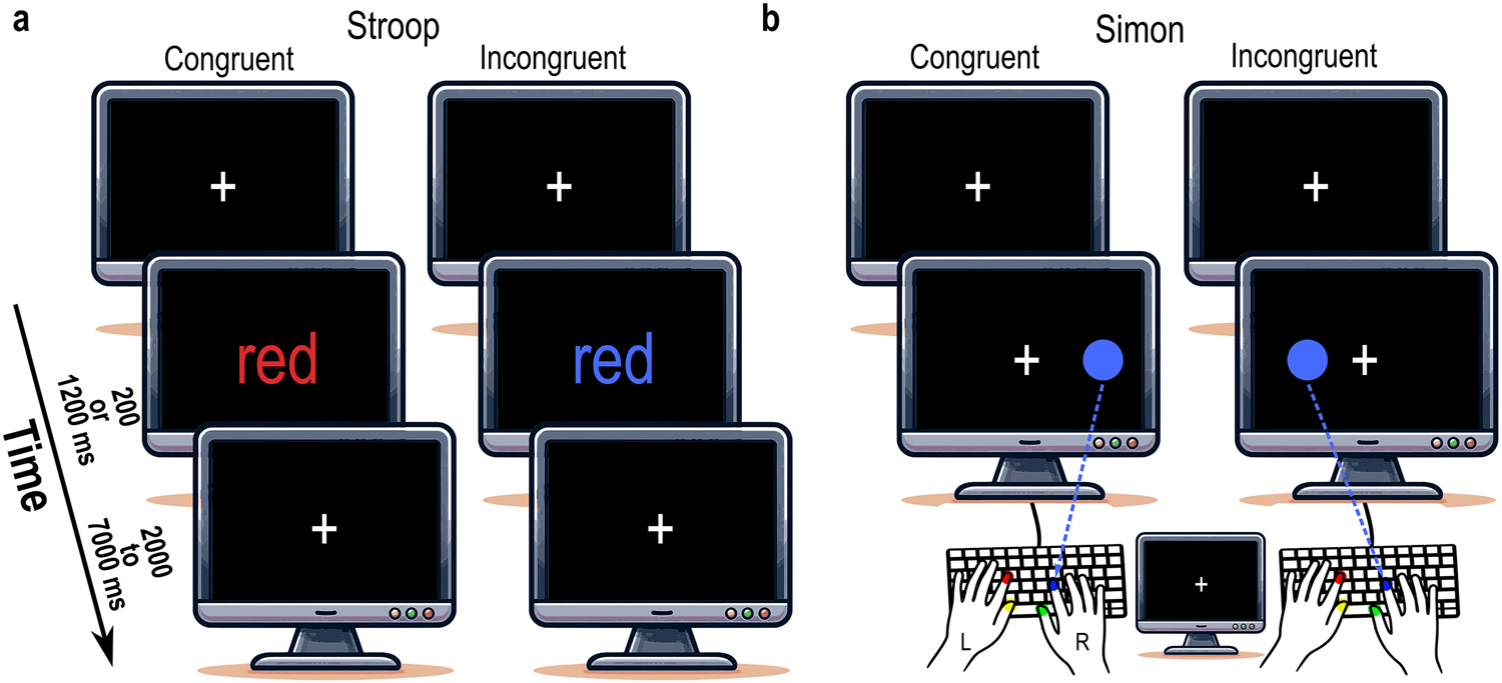
Task designs for EEG-fMRI dataset. (**a**) Stroop task: Participants identify the ink color of congruent and incongruent color words, ignoring the words' meaning. (**b**) Simon task: Conflict arises from incongruent stimulus–response mappings, where participants indicate the ink color of a laterally presented dot, ignoring its location. In both tasks (**a** and **b**), congruent trials entailed no response conflict, while incongruent trials involved semantic mismatch or contralateral stimulus location. Stimuli were displayed for 200 ms or 1200 ms, with a response window of 1200 ms. A fixation cross was present, and the inter-trial interval was randomly selected within the range of 2000 to 7000 ms.

On average, in the simultaneous EEG-fMRI dataset, individuals correctly responded (i.e., Hit) in 204 (standard deviation = 15) incongruent trials and incorrectly responded (i.e., Error) in 21 (standard deviation = 13) incongruent trials, Fig. 1a,b. The omission trials (i.e., failure to respond to a stimulus within a given time limit) were excluded from the analyses.

**Figure 2:**
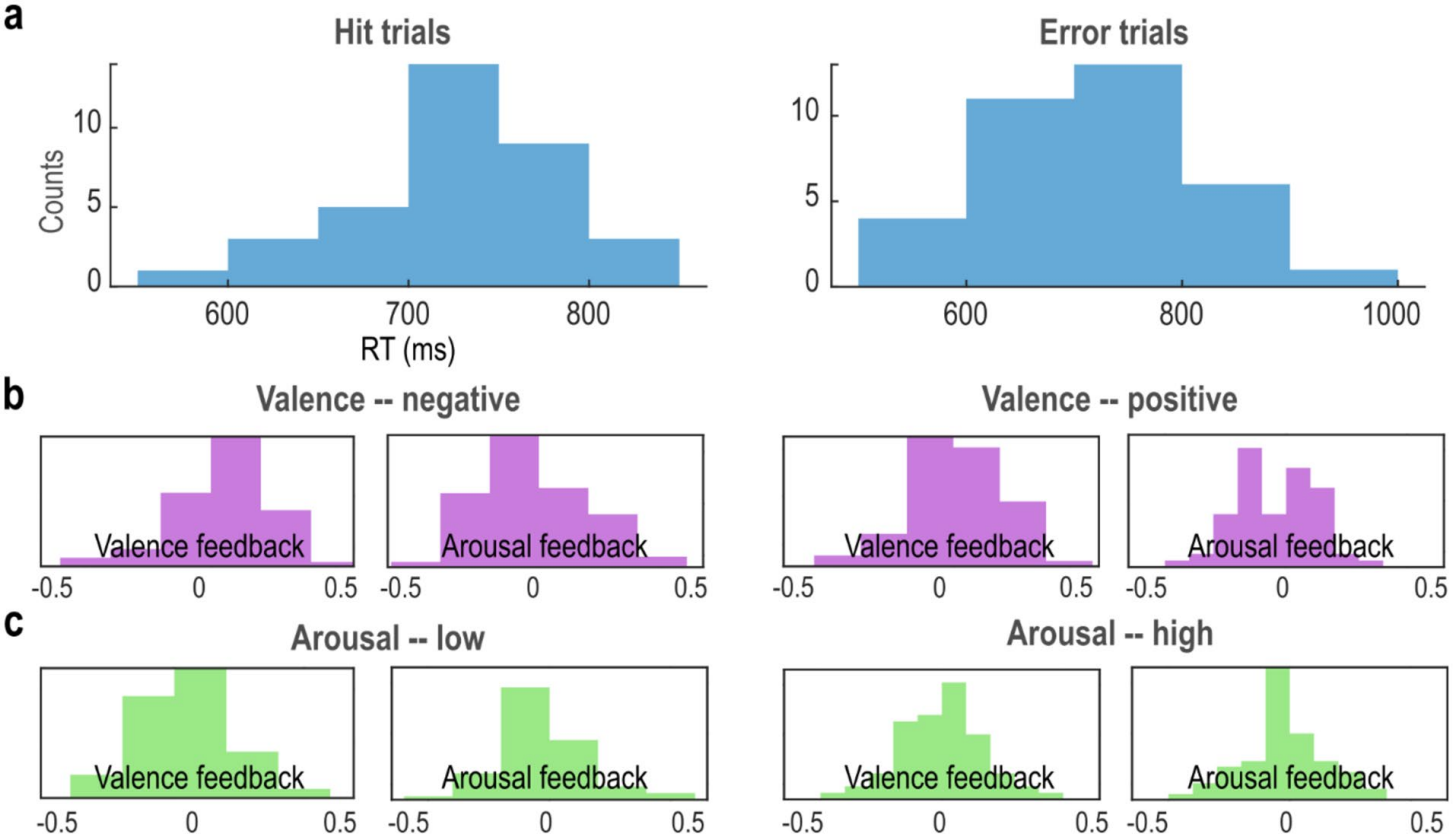
Distributions of behavioral measures for the two datasets. (**a**) The distributions of reaction times (RTs) for Hit and Error trials in the simultaneous EEG-fMRI dataset. (**b**) The distributions of the neurofeedback valence and arousal scores for the negative and positive valence volition conditions in the psychophys-fMRI dataset. (**c**) The distributions of the neurofeedback valence and arousal scores for the low and high arousal volition conditions in the psychophys-fMRI dataset.

The mean (± standard deviation) reaction time (RT) for Hit trials was 726 ± 55 ms, and for Error trials, it was 715 ± 95 ms, Fig. 2a.

There were no direct behavioral measurements in the psychophys-fMRI dataset. Nevertheless, feedback was provided to the participants during both the real and sham sessions, based on the electromyography (EMG) and galvanic skin response (GSR) recordings^17,18^. Please see the Methods section for more details. The neurofeedback for the four conditions (negative and positive valence, low and high arousal) consisted of two scores (i.e., the valence feedback and the arousal feedback) presented as a red dot on a grid. The distributions of these two feedback scores are presented for valence in Fig. 2b (negative valence [mean + standard deviation]: 0.05 ± 0.20, range = [− 0.53, 0.43]; negative arousal: − 0.03 ± 0.21, range = [− 0.47, 0.52]; positive valence: 0.02 ± 0.20, range = [− 0.57, 0.47]; positive arousal: − 0.04 ± 0.16 range = [− 0.45, 0.32]), and for arousal conditions in Fig. 2c (negative valence: 0.05 ± 0.18, range = [− 0.37, 0.55]; negative arousal: − 0.03 ± 0.18, range = [− 0.43, 0.48]; positive valence: 0.00 ± 0.17, range = [− 0.42, 0.46]; positive arousal: − 0.02 ± 0.16, range = [− 0.42, 0.40]), during the real session. The distribution indicates that emotional and arousal volition were not always achieved, raising the question of what the neural underpinning of successful cognitive control, or in this case, affective volition, is—a question we sought to answer in this study. For details about the demographics, refer to Supplementary Tables S1 and S2.

### Predicting conflict errors from preparatory neural activity

**Figure 3:**
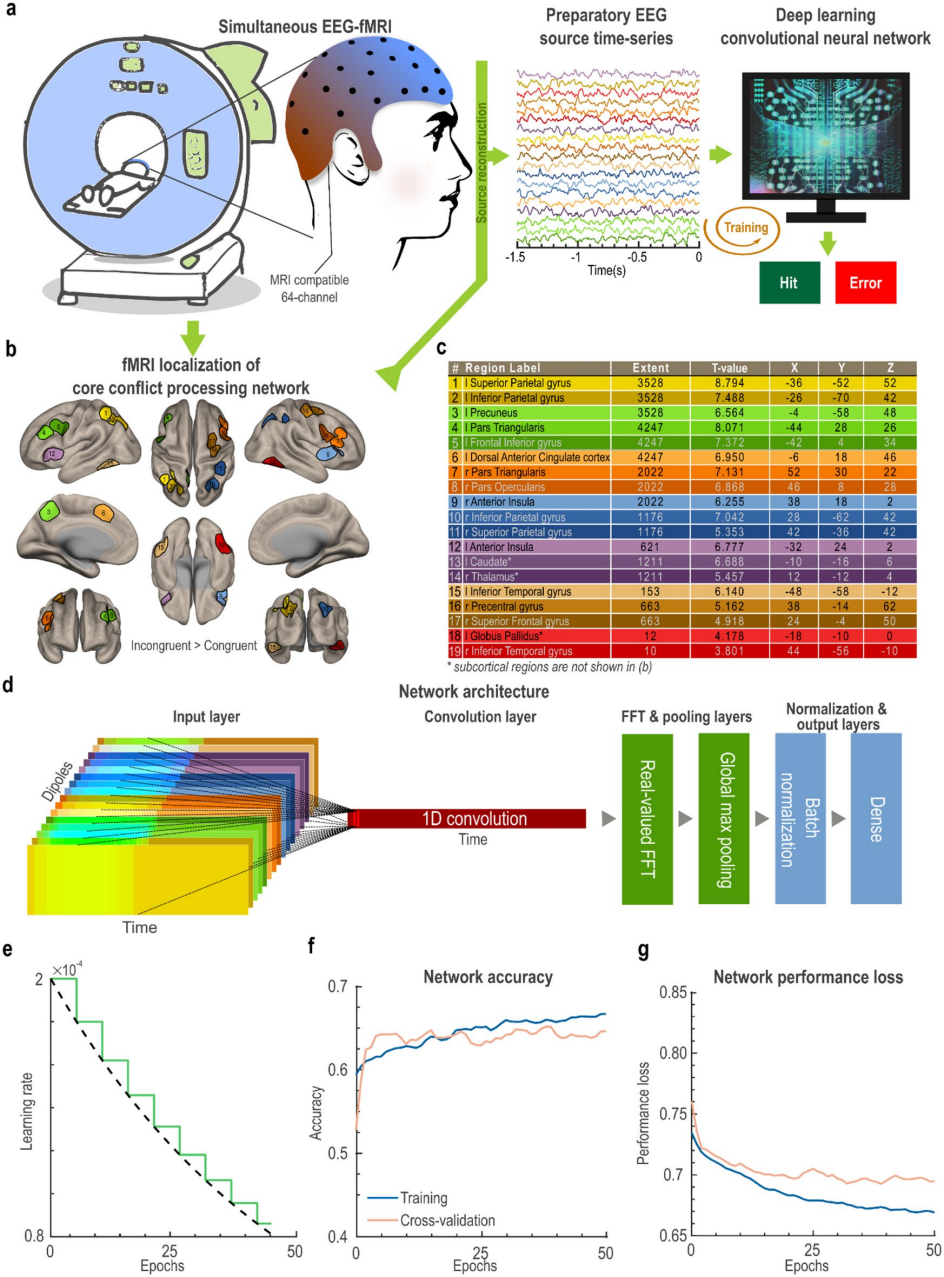
Method summary, network architecture, training, and cross-validation for the primary dataset. (**a**) Concurrent EEG and fMRI data were obtained. Utilizing structural MRI, a four-concentric head model was constructed and employed to reconstruct preparatory EEG signals at (**b**) regions localized by fMRI, showing heightened activation during Incongruent versus Congruent conditions. (**c**) The table includes detailed statistics that outline the specific brain regions identified through fMRI analysis. (**d**) The time-series of the fMRI-guided EEG sources (i.e., Dipoles) were concatenated (Dipoles × Time: 19 × 726) to generate input for the convolutional neural network (CNN). The CNN contains only two layers with trainable parameters: 1D Convolutional and Dense layers. The EEG signals were partitioned into training (60%), cross-validation (30%), and test (10%) sets. The training and cross-validation sets were utilized to train the CNN for discriminating Hit from Error trials. (**e**) The training rate diminishes exponentially in staircase fashion over Epochs. The dotted black line represents the underlying exponential function, while the green line represents the actual training rate per epoch. (**f**) Progression of training and cross-validation accuracies over epochs. (**g**) Progression of training and cross-validation performance losses over epochs.

We used a simultaneous EEG-fMRI dataset to evaluate the potential influence of the PEA of the core cognitive control regions, on conflict processing. A visual representation of our methodology is presented in Fig. 3a. Initially, we identified 19 regions of interest (ROIs) through fMRI analysis by contrasting correct incongruent trials with correct congruent trials (conflict processing), Fig. 3b. Additionally, Fig. 3c provides detailed descriptions of ROIs and corresponding t-statistics for incongruent versus congruent trial activity.

We reconstructed the EEG source time-series of conflict processing ROIs during preparatory intervals (1500 ms pre-stimulus interval: PEA) and subsequently inputted PEA into a CNN to probe if the ongoing activity of core conflict processing network regions could predict the level of conflict error, Fig. 3a. Our previous study demonstrated that a CNN with a straightforward architecture and rigorous regularization terms produces reliable and versatile results^13^. Following this approach, we used a similar strategy and designed a CNN with the minimum number of trainable parameters, a low number of training epochs (i.e., 50 epochs), and large regularization terms, Fig. 3d. The network was trained using Adam optimizer^22^ with an exponential decay learning rate, starting at 2e-4 and decaying every 1000 steps at a rate of 0.90, Fig. 3e. The accuracy (Fig. 3f) and the performance loss (Fig. 3g) across epochs were monitored during the training and cross-validation. Once the training was completed, the network demonstrated an ability to predict the Hit from Error trials (i.e., error processing) significantly above the chance level (i.e., 50%), based on the PEA of 19 core conflict processing network ROIs. At the final epoch, the loss and the accuracy were 0.667 and 67% for training, and 0.700 and 64% for the validation. CNN achieved a test accuracy of 64% (loss = 0.690), a value well above the chance level, indicating a link between the PEA of the cognitive control network and the likelihood of conflict errors.

### Associations between conflict errors and spatio-spectral features of preparatory neural activity

**Figure 4:**
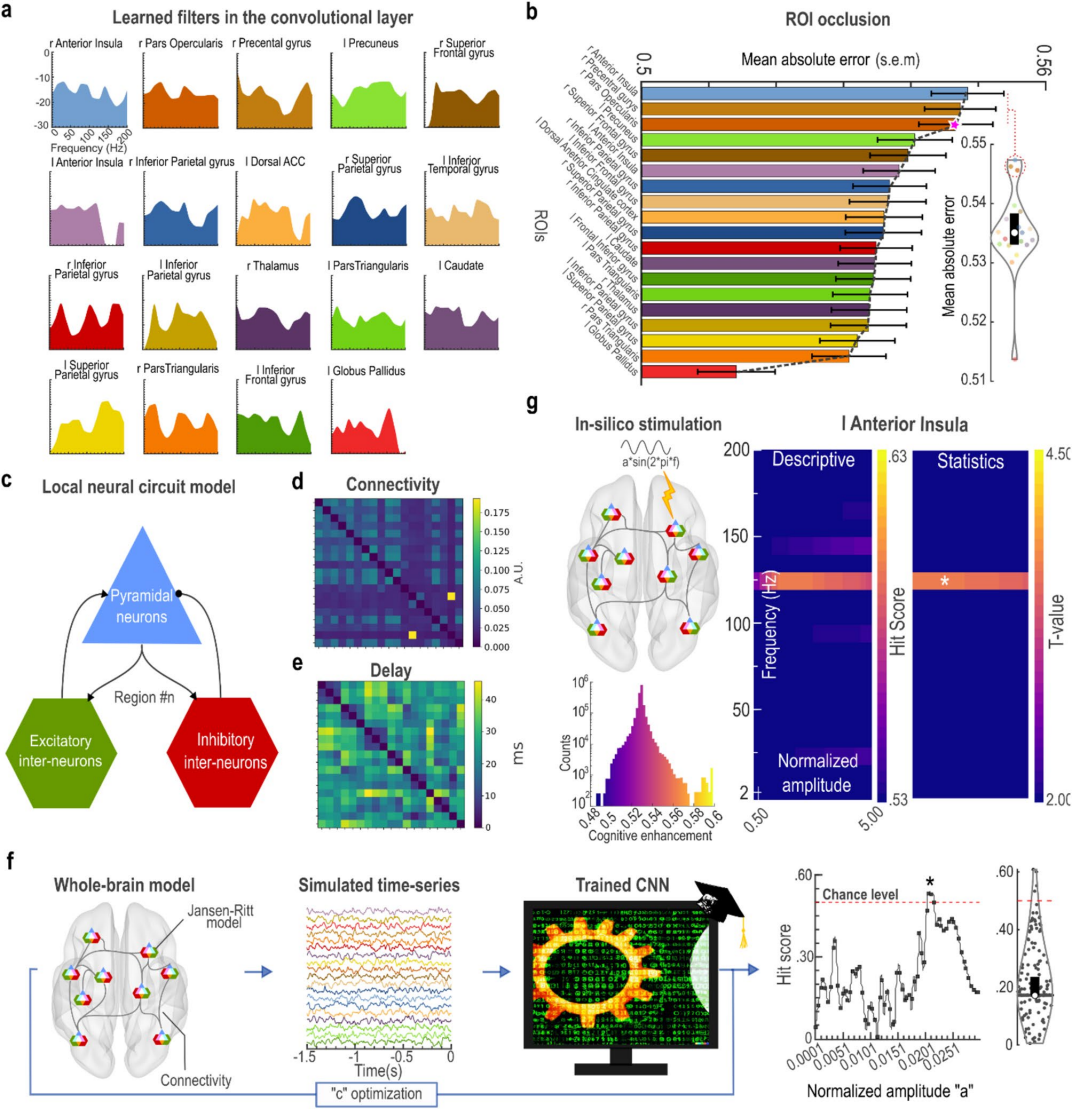
The spatio-spectral preparatory neural signature of conflict errors. (**a**) The spectral density of the convolutional layer for each ROI indicates the involvement of broad-band arrhythmic activity of cognitive control network in conflict processing. (**b**) Bar plots illustrate the mean absolute error (MAE) of prediction, in the occlusion experiment, arranged in a descending order across the ROIs. The error bars represent the standard error of the mean (s.e.m.). A higher MAE for a given ROI indicates a more pronounced adverse effect of that specific ROI’s removal on the prediction of the CNN, and consequently, on cognitive control performance. The point at which the MAE begins to decrease significantly in relation to ROIs is denoted by an asterisk on the third ROI, suggesting that preparatory activity of the initial three ROIs (i.e., the right anterior insula, right precentral gyrus, and the right pars opercularis) exerted the most substantial influence on the CNN’s prediction of cognitive control performance. The inner violin plot shows the distribution of the MAEs, and the scatter plot shows individual MAE values. Interquartile ranges are shown by the black box, and the white dot within the black box indicates the median of MAE. (**c**) The Jansen-Rit neural mass model is comprised of three interconnected neural populations, namely pyramidal projection neurons, excitatory and inhibitory inter-neurons forming feedback loops. The core conflict processing network was modeled as a set of coupled Jansen-Rit models, (**d**) through a connectivity network built from functional connectivity analysis of the simultaneous fMRI dataset. (**e**) The time delays of these connections were estimated based on inter-regional Euclidean distances and a conduction speed of 3 m/s. (**f**) Through a grid-search framework spanning the interval of 0.0001 to 0.05, a global coupling scale factor "c" was systematically fine-tuned to uniformly adjust all connection weights. For every "c" value, a set of simulated EEG signals was produced, which was subsequently fed into the pre-trained CNN to calculate the Hit score. The optimal parameter "c" was defined as the value that yielded a Hit score significantly above the chance level, determined through a 5000-resampling bootstrap test. The distribution of the accuracies associated with tested "c" values is shown in the violin plot in the right panel. The scatter plot within the violin plot shows individual accuracies. The black box represents interquartile ranges, and the white dot within the black box represents the median of the accuracy. (**g**) The Hit score was monitored as a sine wave stimulation was applied to each node of the neural mass model. The stimulation was characterized by a normalized amplitude "a" spanning the range of 0.5 to 5 and a frequency "f" in the range of 2 to 200 Hz. The largest change in the Hit score was found for the left anterior insula (left heatmap). A 5000-randomization test was carried out to create the statistical map (right heatmap) and to assess the statistical significance of improvement in the Hit score followed by each stimulation. Applying stimulation with a frequency of approximately 130 Hz and a normalized amplitude of around 1, targeted at the left anterior insula, led to the most significant improvement in the Hit score. The histogram indicates the distribution of the enhanced Hit scores (i.e., cognitive enhancement) for different stimulation protocols. The warm-colored cluster around 0.60 in the histogram is related to 130 Hz stimulation of left anterior insula.

The objective of the CNN was to maximize the dissociation between Hits and Errors based on the pre-stimulus baseline PEA in the conflict processing network. We were critically interested in identifying the most informative spatio-spectral features for this classification. This was achieved through a combination of real-valued fast Fourier transformation (FFT) and global max pooling layers applied after the convolutional layer in our CNN model, Fig. 3d. To assess the spectral features across the ROIs, we extracted the feature maps produced by the convolutional layer for each ROI and then transformed these feature maps into the Fourier space. This approach allowed us to analyze the frequency characteristics of the PEA and identify key spectral features for classification. We found that the spectral characteristics of the predictive PEA were not restricted to a particular frequency band, but instead they were driven by arrhythmic neural signals across a wide frequency range, Fig. 4a. This points to the crucial role of preparatory arrhythmic broadband neural dynamics for predicting prospective cognitive control performance.

Next, we applied an iterative occlusion experiment to understand the importance of different ROIs’ PEA for the CNN’s decision-making process. The occlusion procedure consisted of a step-by-step blocking of each ROI, followed by observing how this impacts the CNN’s prediction of prospective cognitive control performance based on preparatory activity in the remaining ROIs. Importantly, we employed an independent test sample (n = 789; Hits: 715, Errors: 74) to ensure that the analysis was conducted without any risk of double-dipping. The systematic perturbation of the core cognitive control regions and assessment of CNN’s performance aided us in localizing the regions with the most crucial information for prediction of the conflict errors (i.e., largest predictive value). The loss in performance of the CNN was quantified as the mean absolute error (MAE) and sorted in descending order, displaying a clear inflection point, commonly referred to as the "knee", manifested at the third ROI (marked with an asterisk mark in Fig. 4b). This observation suggests that the first three ROIs, namely the right anterior insula cortex (MAE = 0.5473; *t* = 1.73, *p* = 0.049), the right precentral gyrus (MAE = 0.5463; *t* = 1.57, *p* = 0.099) and the right pars opercularis (MAE = 0.5456; *t* = 1.48, *p* = 0.152) contained the most distinctive information for the prediction of conflict errors (inner violin plot of Fig. 4b). The significance level (i.e., *p*-value) was determined by running a permutation test with 5000-randomization. Although the right anterior insula cortex had the highest predictive values for the CNN’s decision making (i.e., dissociation of Hit from Error), empirical or simulated intervention data were required to ascertain whether any intervention targeting these regions could result in the cognitive control enhancement. Therefore, we further modeled the effect of external stimulation on every ROI and assessed the Hit score generated by the CNN.

### High frequency stimulation of the left anterior insula for cognitive enhancement

We integrated our CNN model with a neural mass model to further assess the effect of neurostimulation on functioning of conflict processing regions. We constructed a model of conflict processing network where 19 ROIs, each modelled by a Jansen-Rit neural population model^23^, Fig. 4c, interacting through a network characterized by functional connectivity measures derived from simultaneous fMRI data, Fig. 4d. Time delays in the interconnections were estimated based on the Euclidean distance among ROIs and a conduction speed of 3 m/s, Fig. 4e. Drawing inspiration from neural network generative models, we integrated the Jansen-Rit neural mass model serving as the generator, with the CNN in the role of the discriminator. We used a grid-search framework with 200 steps, spanning the range of 0.0001 to 0.05, to estimate the optimal value for the global coupling scale factor "c", aiming to achieve a Hit score significantly above the chance level. The neural mass model with the choice of c = 0.021 produced the highest significant Hit score (53%), *t* = 3.43, *p* = 0.005, determined through a 5000-resampling bootstrap test, Fig. 4f. Notably, in order to avoid overfitting and to minimize computational cost, our parameter optimization focused on the coupling scale factor "c", while keeping all other parameters fixed at their default values as defined for the Jansen-Rit model^23^. Please also see Table 2 under the Material and Methods.

Finally, we examined the impact of in silico stimulation targeting different ROIs in the core cognitive control network, for the improvement of Hit score. Stimulation was modeled as a sine wave, with a normalized amplitude "a" ranging from 0.5 to 5, and a frequency "f" ranging from 2 to 200 Hz, each divided into 20 intervals. The Hit score was monitored as stimulation was applied to each node of the coupled Jansen-Rit neural mass model. Applying neurostimulation at approximately 130 Hz with a normalized amplitude around 1, targeted to the left anterior insula, led to a significant enhancement in the Hit score (*t*(18) = 4.05, *p* = 0.0007, determined by 5000-resampling randomization test; Fig. 4g). The simulated stimulation effects for all other regions can be found in Supplementary Fig. S1.

### Real-time intrinsic modulation of the anterior insula during volitional control

As a follow-up to our model-based findings, we utilized a neurofeedback dataset where real-time measurement of neural activity with fMRI was used as a source of feedback to guide volitional control (intrinsic neuromodulation) of affective processing^17,18^. To this end, subsequent to the presentation of an affective goal, a visual emotion regulation instruction appeared (in the form of the word "FEEL") accompanied by either real or sham neurofeedback, unbeknown to the participants. This neurofeedback signified brain activity in relation to the elicited emotion. Participants drew upon pre-prepared mental strategies to self-induce the experience of that emotion according to the desired goal.

**Figure 5:**
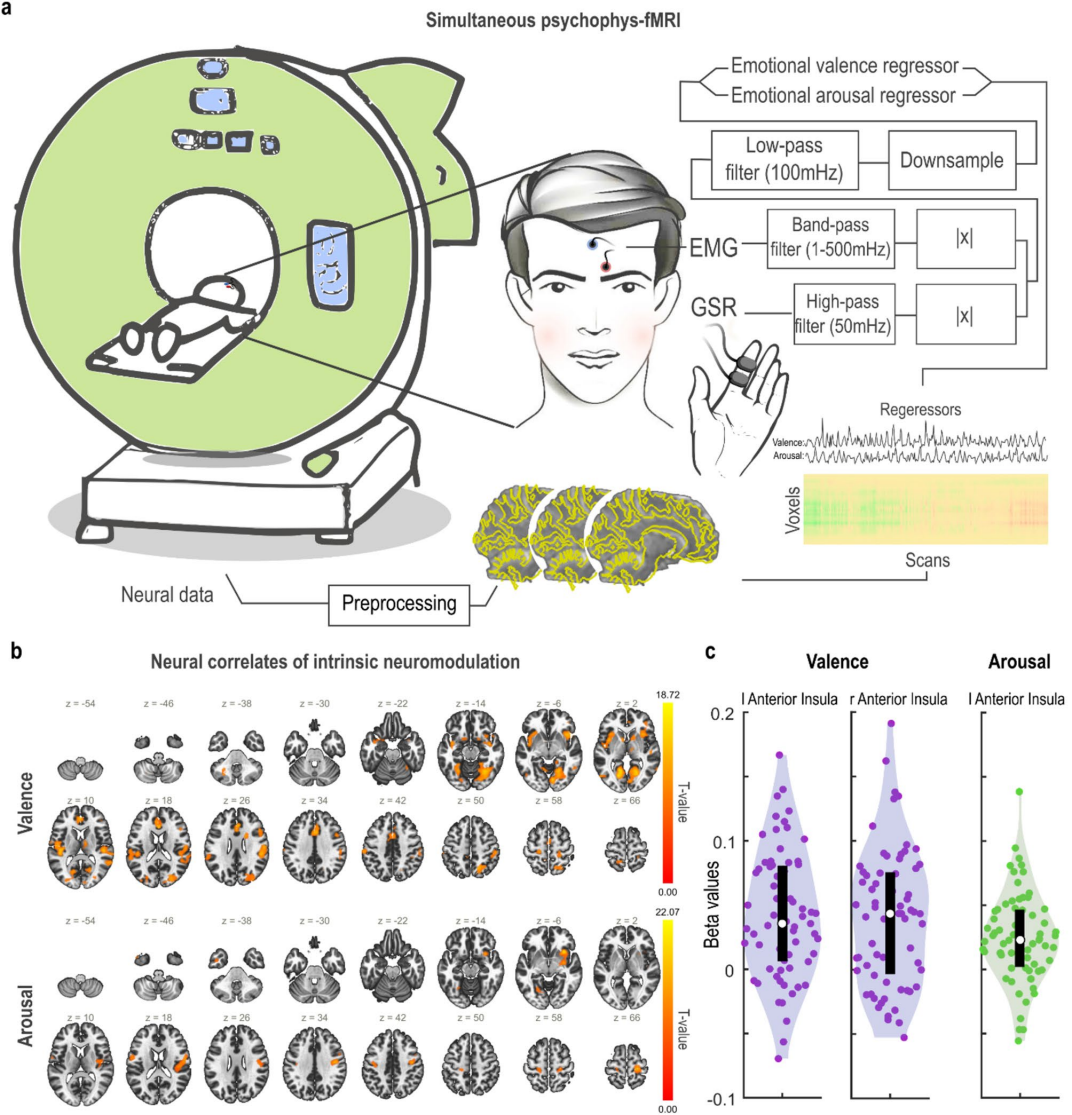
The anterior insula activity in relation to real-time neurofeedback-guided volitional affect regulation. (**a**) Summary of the analysis method to identify neural correlates of intrinsic neuromodulation in the secondary dataset. Psychophysiological measures of valence (facial electromyography, EMG) and arousal (galvanic skin response, GSR) were recorded concurrently with the fMRI data collection. After preprocessing and motion/physiological noise correction, a General Linear Model was formulated to determine brain regions where BOLD activation during volitional control correlated with each psychophysiological measure (either real-time emotional valence or arousal regressor). (**b**) Significant clusters in bilateral insula were found to be associated with emotional valence (facial EMG, upper panel) and arousal (GSR, lower panel). FDR-corrected (*p* < 0.05 cluster-level threshold) effect sizes (i.e., t-values) are color-coded and superimposed on the anatomical MNI template. (**c**) Violin plots depict effect distribution in the anterior insula; individual beta values are shown as scatter plots within the violin plots, with interquartile ranges represented by black boxes and the median indicated by white dots.

We specifically sought to investigate whether volitional control was related to the intrinsic neuromodulation of anterior insula. To assess the neural basis of volitional control in real-time, we utilized simultaneous fMRI and psychophysiological data, including EMG activity from the corrugator supercilii muscles as a measure of emotional valence and GSR data capturing emotional arousal^24^, Fig. 5a. The fMRI data underwent standard preprocessing, followed by CONN’s standard denoising pipeline^25^. Notably, the pre-processed and down-sampled EMG and GSR were used to create the regressors of interest for emotional valence and arousal, Fig. 5a.

To determine brain regions co-fluctuating with emotional regressors in real time, we first assessed the Fisher’s Z-transformation of the bivariate correlation coefficients from a weighted general linear model (GLM)^26^ that characterized the association between the BOLD and emotional regressors during the "FEEL" interval, at the individual-level for the real session. At the group-level, we found that the neural activity of the bilateral insula (right: *t(66)* = 5.66, *p*_*FDR cluster corrected*_ < 1e-6, k = 1668; left: *t(66)* = 5.91, *p*_*FDR cluster corrected*_ < 1e-6, k = 2924) among other regions (please refer to Supplementary Table S3 for specifics), was significantly associated with the emotional valence regressor (Fig. 5b, upper panel). However, for the emotional arousal, we found that among other regions (please see Supplementary Table S4 for details), only the right insula cortex (*t(66)* = 6.10, *p*_*FDR cluster corrected*_ < 1e-6, k = 1822) was significant (Fig. 5b, lower panel). We additionally extracted the peaks of individual beta values from significant clusters in the left and right anterior insula for emotional valence, and in the left anterior insula for emotional arousal conditions Fig. 5c. Assessing the distribution of the beta values confirmed the observed group-level statistics.

## Discussion

This study represents a multifaceted approach to investigating the intricate relationship between pre-stimulus baseline PEA and proactive cognitive control using two publicly accessible, but unique, multimodal datasets, delving into sub-trial brain activity levels. We proposed a neuroimaging-informed CNN model, specifically designed to capture the spatio-spectral characteristics of the pre-stimulus baseline PEA for predicting prospective cognitive control performance. Through the integration of our CNN model with an occlusion experiment, we elucidated the significance of distinct brain regions and their spectral characteristics in the model's decision-making process. We found that preparatory arrhythmic broadband neural dynamics, rather than frequency-specific rhythms, in the right anterior insula, right precentral gyrus, and the right opercular part of inferior frontal gyrus (posterior ventrolateral prefrontal cortex), are highly predictive of prospective cognitive control performance.

Furthermore, we investigated the effects of neuromodulation on cognitive control, both theoretically and experimentally. Theoretically, we utilized neural mass modeling as a generative model coupled with the trained CNN. Experimentally, we assessed the effects of intrinsic neuromodulation via neurofeedback-guided volitional control. We found that high-frequency (130Hz) stimulation of the left anterior insula provides significant cognitive enhancement, notably reducing conflict errors, despite the greater predictive value of the right anterior insula's preparatory activity for prospective inhibition and attention control. This implies that effective neurostimulation targets may differ from regions showing the relevant biomarker activity, highlighting the intricate nature of cognitive networks. We indirectly validated this theoretical finding by analyzing concurrent psychophysiological and fMRI data collected during neurofeedback-guided volitional control, which allowed for the evaluation of intrinsic neuromodulation. Specifically, we observed that the left anterior insula activity was intrinsically modulated in real-time by volitional control of emotional valence, with no corresponding effect observed for self-regulation of emotional arousal.

Our findings further highlight the central role of the anterior insula in orchestrating proactive cognitive control processes, positioning it at the top of the hierarchy for cognitive control, yet left and right anterior insula seem to be operating at different levels in the cognitive control hierarchy. Particularly, the left anterior insula seems to have a pivotal role in fundamental cognitive control mechanisms, supporting interference resolution and conflict-related decision making, regardless of the eventual choice. On the other hand, the predictive power of PEA in the right anterior insula, right precentral gyrus, and the right opercular part of inferior frontal gyrus (posterior ventrolateral prefrontal cortex) may underscore their role in elaborate attentional processing, active goal maintenance, planning and preparation of target-specific responses, stimulus processing and categorization, performance monitoring, and updating the action plans.

We found that preparatory arrhythmic broadband neural dynamics, rather than frequency-specific rhythms, play a crucial role in proactive cognitive control. This highlights the importance of non-oscillatory neural processes in predictive monitoring for prospective cognitive control performance. EEG research suggests that broadband activity is indicative of asynchronous neuronal firing, whereas synchronization activity appears to be more implicated in collective states devoid of new information creation^27,28^. Theoretical evidence also supports that desynchronized brain activities, marked by asynchronous and irregular activity, enhance stimulus responsiveness and facilitate the integration of multiple external inputs into intrinsic brain activity^20,29^. Of note, we previously found that patients with lower global synchrony were more responsive to the neurostimulation^20^. Additionally, evidence indicates that adaptive cognitive control is achieved through intermittent macroscopic neural dynamics,  characterized by the broken symmetry of spatiotemporal patterns, which are found in broadband activity^28,30^. The dynamic, non-sinusoidal nature of broadband brain activity poses challenges for traditional Fourier-based analyses, and while alternative methods such as empirical mode decomposition have been suggested, issues like mode mixing persist, especially in noisy brain signals with transient bursts^31^. In this study, we introduced a refined spectral decomposition approach that enhances conventional Fourier-based analysis. We used a CNN to apply a data-driven, latent-basis transformation to learn filters, which helped us uncover obscured broadband activity masked by simultaneous changes in specific rhythmic patterns or artifacts.

Most cognitive control electrophysiological studies focus on grand average post-stimulus baseline-normalized ERPs, overlooking pre-stimulus preparatory activities, limiting our understanding of the role of the PEA's temporal dynamics in predicting prospective cognitive control performance. Despite its limitations, the conventional approaches have provided insights into early sensory processing and reactive control mechanisms following stimulus presentation. Post-stimulus activity in the bilateral anterior insula during the Go/No-Go discrimination task has been associated with perceptual decision-making, and it has been proposed to serve as a potential generator of distinct prefrontal ERPs, associated with various cognitive control stages^3,12^. Moreover, there is evidence that the left anterior insula activity—being not modulated by stimulus category and having a peak almost coincident with the response—might reflect the time at which the decision is made (the decision-making process), independently of the ultimate choice (action or inhibition), however, the right anterior insula activity is influenced by the stimulus category, suggesting its implication for stimulus categorization and performance monitoring^12^. The anterior insula, located at the crossroads of higher cognition and motivation pathways, integrates interoceptive information with emotional, cognitive, and motivational signals. It supports unique subjective feeling states and helps determine the importance of stimuli based on internal states, effectively allocating cognitive resources^9^. The anterior insula has also recently been proposed as the gatekeeper to executive control^32^.

In this study, we sought to address the limitations of current neuroimaging techniques, particularly their low spatial or temporal resolution, by integrating multimodal neuroimaging data (mainly simultaneous EEG-fMRI), with computational modeling, including both CNN and neural mass modeling. Recognizing the inadequacy of statistical associations between neural activity and behavior alone in revealing underlying mechanisms, we employed a combination of counterfactual conditions and neurostimulation to establish causality. To this end, we employed both in-silico and empirical interventions, enhancing our study and advancing toward causality. In our system-level model of the inhibitory attentional control network, each ROI was represented by a Jansen-Rit model, interconnected through a realistic connectome^19,33^, derived from functional connectivity analysis of simultaneous fMRI data. A coupling factor was incorporated to uniformly scale inter-regional connectivity measures across the model. Optimization of this factor was achieved by minimizing the CNN's loss, serving as our cost function. While exploring Jansen-Rit model parameters could potentially enhance model performance by reducing the CNN's prediction loss, this was avoided due to the risk of overfitting. When coupled together, collective behavior of interacting neural mass models has been shown to reproduce key features of brain dynamics, providing a theoretical foundation for investigating various stimulation parameters and devising an optimal neurostimulation protocol. Thereby, we could assess the impact of in silico stimulation targeting different ROIs within the core cognitive control network on enhancing inhibitory attentional control. This was measured by observing increased Hit scores. Our findings revealed that applying 130 Hz stimulation to the left anterior insula cortex provided the most significant increase in the likelihood of avoiding conflict errors. We indirectly and experimentally validated our theoretical finding regarding the causal role of the left anterior insula in cognitive control using an intrinsic neuromodulation dataset. Still, future neurostimulation studies are crucial for validating this hypothesis and evaluating the target engagement, and the feasibility of cognitive enhancement. While deep brain stimulation offers precise high-frequency neurostimulation, its limitations and risks necessitate a prioritization of non-invasive methods like transcranial alternating current stimulation^33^.

Future studies may benefit from incorporating advancements in model optimization. For instance, Bayesian inference methods can provide parameter estimates with associated uncertainties^34^, and adaptive Hamiltonian Monte Carlo can facilitate efficient exploration of parameter spaces^35^. Introducing heterogeneity to local oscillator models across brain regions, while avoiding overfitting, could be achieved using data-driven dimension-reduction techniques and hierarchical optimization methods such as Hierarchical Alternating Least Squares^36^. Normalizing Flows can transform simple initial distributions into complex target distributions, enhancing sampling and density evaluation capabilities while preserving total probability and incorporating domain knowledge^37^. Furthermore, the framework of variational autoencoders can address the phenomenon of posterior collapse, where the generative model learns to ignore some latent variables, causing the approximate posterior to closely match the prior distribution along these dimensions^38^.

To summarize, we conducted comprehensive computational and experimental research to better understand the link between pre-stimulus PEA and proactive cognitive control, as well as to explore the potential of neurostimulation for cognitive enhancement. Our findings highlight the predictive power of pre-stimulus arrhythmic broadband PEA in the right anterior insula, right precentral gyrus, and the right opercular part of inferior frontal gyrus (posterior ventrolateral prefrontal cortex) for inhibitory attentional control. Furthermore, we provided evidence that applying high frequency neurostimulation to the left anterior insula has the potential to improve the inhibitory attentional control aspect of cognitive control. Our study offers new insights into the neural processes underlying proactive cognitive control and suggests potential therapeutic approaches.

## Methods

### Data and participants

We used two open access databases to pursue the aims of the current study. The primary dataset contains simultaneous EEG-fMRI recording that is shared by Beldzik and colleagues on Open Science Framework (OSF, https://osf.io/cx8a9/)^16^. The EEG-fMRI data was collected from 37 individuals (mean age, 22 ± 3 years; 22 women). All participants were right-handed, determined by the Edinburgh Handedness Inventory^39^, Supplementary Table S1, had no history of physical or psychiatric disorders, and were drug-free. The second dataset (i.e., psychophys-fMRI dataset) shared by Fialkowski and colleagues (OpenNeuro Dataset ds003831)^17,18^, includes concurrent EMG, GSR, and fMRI data collected from 71 participants during real-time guided affect self-regulation (i.e., intrinsic neuromodulation of core effect) task. The data of 4 individuals were removed due to low signal quality, and therefore the final sample included 67 individuals (mean age, 39 ± 14 years, 47 women), Supplementary Table S2. Moreover, all participants provided written informed consent and all the aspects of the two studies were in accordance with the guidelines of the declaration of Helsinkin^16–18^.

### fMRI data acquisition and task

For the primary EEG-fMRI dataset, the MRI scans were collected on a Siemens 3T scanner (Magnetom Skyra, Siemens) with a 64‐channel head/neck coil. The structural T1 weighted scans were acquired (TR = 2300 ms, TE = 2.98 ms, flip angle = 9°, voxel size = 1 × 1 × 1.1 mm^3^) together with functional T2*‐weighted functional scans using a whole‐brain echo-planar imaging (EPI) pulse sequence (TR = 1800 ms, TE = 27 ms, flip angle = 75°, FOV = 224 × 224 mm^2^, 3.5 mm isotropic voxel, GRAPPA acceleration factor 2, and phase encoding A/P)^16^. Participants completed two types of conflict-inducing tasks, namely Stroop and Simon tasks. The Stroop task involved a stimulus with four color names printed in Polish, while the Simon task involved a dot presented laterally to a fixation sign. Participants were instructed to indicate the color by pressing a button on a response grip. Response conflict was present on incongruent trials, where the target response was a semantic mismatch or contralateral to the stimulus location, and no conflict was present on congruent trials. The stimulus was presented for either 200 ms or 1200 ms, with a 1.2 s response window. A fixation sign was present throughout the experiment, except for the stimuli presentation period in the Stroop task. The intertrial time interval (ITI) was randomly drawn from a uniform distribution, resulting in an average of 3.5 s. Each task consisted of 420 trials which were presented in two blocks with 50 and 20% congruency rates. Four blocks were counterbalanced among individuals, but task types remained interleaved. The design controlled for the task type (Stroop vs. Simon), conflict (congruent vs. incongruent), congruency rate (50 vs. 20%), and stimulus duration (short vs. long).

For the psychophys-fMRI dataset, the structural and functional neuroimaging data were acquired on a Philips 3T Achieva X-series scanner (Philips Healthcare, Eindhoven, The Netherlands) with a 32-channel head coil. The structural T1-weighted scans were collected (TR = 80,844 ms, TE = 3.7010 ms, flip angle = 8°, voxel size = 0.94 × 0.94 × 1 mm^3^) together with T2*-weighted functional scans with the help of whole-brain EPI pulse sequence (TR = 2000 ms, TE = 30 ms, flip angle = 90°, FOV = 240 × 240 mm^2^, 3 mm isotropic voxel)^17^. During a 10-min task, participants engaged in real-time guided affect self-regulation trials. Each trial commenced with a 4-s display of an affective goal, including instances of positive valence, negative valence, high arousal, and low arousal. Following this, self-regulation instructions were provided (indicated by the term "FEEL") for a duration of 10 s, coupled with either real (based on subject’s brain activity) or sham (unrelated to the subject’s brain activity) neurofeedback (depicted as a red dot on a grid spanning valence and arousal scores). This phase lasted for 4 s and was randomly counterbalanced among participants. The sham neurofeedback was visually realistic and derived from a high-performing subject’s brain activity during the pilot phase of the experimental design.

### MRI pre-processing and denoising

A flexible preprocessing pipeline^26^ was applied to the structural and functional (f)MRI of the two datasets. This included realignment, slice timing correction, outlier detection, segmentation, and normalization to the MNI-space. A 6-parameter rigid body transformation was used to align the functional data to a reference image (i.e., the first scan of every session)^40^, to account for the head movements throughout the scan. We used ART^41^ to identify potential outliers by looking for framewise displacements greater than 0.9 mm or global BOLD signal changes greater than 5 standard deviations^42,43^. A new reference scan for each participant was calculated by averaging the scans and removing the discovered outliers. Next, functional and anatomical scans were normalized to standard MNI template, and segmented to gray matter, white matter, and CSF tissue classes^43,44^. Finally, functional data was smoothed using spatial convolution with a Gaussian kernel of 8 mm.

After the pre-processing steps were completed, functional data was denoised using a standard denoising pipeline^26^. In a nutshell, the denoising pipeline included regressing out potentially confounding effects, including 5 principal components of white matter and 5 principal components of the CSF, realignment parameters, and their derivatives, detected outlier scans, as well as session and task effects, and the linear trends. Noise components of white matter and CSF were estimated using CompCor^45^. The data was then bandpass filtered. All the pre-processing and data denoising steps were carried out in the CONN toolbox within MATLAB 2022a (The MathWorks, Natick, Massachusetts 2022).

### Localizing the core cognitive control network

The primary fMRI dataset was used for the activation-based analysis of conflict processing (correct incongruent versus correct congruent trials) in a GLM setting. The core cognitive control network regions (i.e., in the sense of blobs consisting of at least 10 voxels exhibiting higher activity for the incongruent than congruent conditions) were identified using a two-sample t-test analysis (*p* < 0.001; df = 34). Subsequently, the center coordinates of those regions were used to precisely position the dipoles for EEG source reconstruction.

### EEG pre-processing

The pre-processing pipeline of the 64-channel EEG data included re-referencing all channels’ data to the common average, applying lowpass filtering with a cutoff frequency of 200 Hz, and removing the line noise by notch filtering at 50 Hz, 100 Hz, and 150 Hz. We further removed ocular, muscle, and scanner artifacts by applying the FastICA algorithm to carry out independent component analysis (ICA). Noisy components were manually detected and removed (Supplementary Fig. S2). The artifact-free EEG was projected back from the ICA space to the data space using the noise-free independent components. Given that the focus of our study was PEA, we only included the trials with the onset that occurred at least 1500 ms after the previous trial’s response, in order to achieve sufficiently long preparatory intervals. The covariance of preparatory signals was averaged and stored for source reconstruction. All the pre-processing steps were carried out using the FieldTrip toolbox^46^ in MATLAB 2022a (The MathWorks, Natick, Massachusetts 2022).

### Source time-series reconstruction

To this end, the fMRI analysis was used to localize brain regions with significantly higher activity in the incongruent compared with the congruent condition (i.e., implicated for successful conflict processing). The high spatial resolution of the fMRI aided us in localizing anatomically accurate and functionally relevant dipoles in the MNI coordinate for EEG source reconstruction. The source reconstruction, using the eLORETA algorithm^47^, was carried out using the source model and the individualized concentrical spherical head models^48^.

Individual T1 scans were segmented into four different tissues including scalp, skull, gray matter, and white matter. Hence, 4 concentric spheres, one per tissue, were considered with conductivity of 0.43, 0.01, 0.33, and 0.14, respectively^48^. The standard 10–20 electrodes’ position was used and realigned to fit the individual head model using the affine transformation. Next, the dipole coordinates that derived from the fMRI analysis in the MNI coordinates were transformed to individual native space to correspond with the realigned electrode positions and the head model.

The eLORTEA method with the regularization parameter (lambda) of 15% was used to solve the inverse problem and estimate the parameters of the common filter to map the scalp potentials to source time-series using the covariance matrix of preparatory EEG (i.e., -1500ms to -50ms). The dipoles’ (i.e., EEG sources') time-series were reconstructed along the Cartesian axes (i.e., ${\overrightarrow{Dipole\left(t\right)}=d}_{x}\left(t\right) \widehat{i}+ {d}_{y}\left(t\right) \widehat{j}+ {d}_{z}\left(t\right)\widehat{ k}$) and later projected to their first principal component using the singular value decomposition. The projected dipole time-series were unity normalized and constituted the input of the CNN.

### Convolutional neural network

Our approach differed from conventional MEEG analysis in that we did not rely on pre-defined canonical brain oscillation bands. Instead, a CNN was trained to learn the frequency characteristics of the cognitive control network nodes during the preparation time.

### Network architecture

We used a CNN to predict the performance of individuals (Hit vs. Error) on incongruent trials by analyzing the preparatory temporal dynamics (i.e., PEA) of the core cognitive control network regions. The CNN used a 1D convolutional layer to identify key patterns from the time-series of 19 dipoles placed in conflict processing areas. After the convolutional layer, real-valued FFT and global max pooling layers were applied to ensure effective learning of the spectral features by the convolutional layer. As in our previous study, our goal was to achieve feature maps that were robust and physiologically interpretable while minimizing the number of trainable parameters in the network. Therefore, we deliberately avoided including any hidden layers after the convolutional layer. The output of the global max pooling layer was processed through a batch normalization layer with a batch size of 25 and then a dense layer. The parameters of the CNN can be found in Table 1.

**Table 1:** The CNN layers and parameters.

Layer	Type	Output shape	Kernel	Stride	Param #
1	Input	(None, 726, 19)	–	–	0
2	Convolution 1D	(None, 695, 1)	32	1	609
3	Real-valued fast Fourier transform	(None, 348, 1)	–	–	0
4	Global maximum pooling	(None, 1)	–	–	0
5	Batch normalization		–	–	2
6	Dense	(None, 2)	–	–	4
		Total (non-trainable)			615 (2)

### Network training, cross-validation, and testing

We randomly partitioned the data into training, cross-validation, and testing subsets, with proportions of 60%, 30%, and 10%, respectively. This resulted in 4,255 samples for training, 2,177 samples for cross-validation, and 710 samples for testing. We utilized the Adam optimizer to train the network for 100 epochs. However, we employed an early termination criterion based on the saturation of the decrease in loss. Consequently, the network was trained for 50 epochs before reaching this criterion. Throughout the training process, we employed an exponentially declining learning rate, which was defined as a function of the number of steps:

1 $$learning \;rate\left( {step} \right) = 2 \times 10^{ - 4} \times 0.90^{{\left\lfloor {\frac{step}{{1000}}} \right\rfloor }}$$

where $\left\lfloor . \right\rfloor$ represents a round-down operation and therefore the learning rate descends in a staircase manner, Fig. 3e. Accordingly, in the later stages of the training, more precise weight adjustments were achieved. It is important to mention that in order to mitigate potential bias arising from an imbalanced number of samples between the "Hit" and "Error" categories, we incorporated class weights (Hit = 5.34, Error = 0.55) when estimating accuracy and performance loss.

### Neural mass modelling of the cognitive control network

We constructed the computational model of the core cognitive control network as a system of coupled Jansen-Rit models^23^. The Jansen-Rit model is a neural mass model consisting of a neural population composed of three interconnected neural subpopulations, namely pyramidal projection neurons, excitatory interneurons, and inhibitory interneurons forming feedback loops Fig. 4c. The local dynamic of each ROI is defined as follows:

2 $$\left\{\begin{array}{c}{\dot{y}}_{0}= {y}_{3}\\ {\dot{y}}_{3}=A a S\left({y}_{1}-{y}_{2}\right)-2a{y}_{3}- {a}^{2}{y}_{0}\\ {\dot{y}}_{1}= {y}_{4}\\ {\dot{y}}_{4}=Aa\left[p\left(t\right)+ {\alpha }_{2}J+S\left({\alpha }_{1}J{y}_{0}\right)\right]-2a{y}_{4}- {a}^{2}{y}_{1}\\ {\dot{y}}_{2}= {y}_{5}\\ {\dot{y}}_{5}=Bb\left[{\alpha }_{4}JS\left({\alpha }_{3}J{y}_{0}\right)\right]-2b{y}_{5}- {b}^{2}{y}_{2}\end{array}\right.$$

where y_j_ values are the state-variables (i.e., y_0,_ y_1,_ and y2 are population-average membrane potential of pyramidal cells, excitatory interneurons, and inhibitory interneurons, respectively) and *S(.)* is a sigmoid function defined as follows:

3 $$S\left(v\right)=\frac{2 {\nu }_{max}}{1+ {e}^{r( {v}_{0}- v)}}$$

We used default parameter values of the Jansen-Rit model, as listed in Table 2.

**Table 2:** Fixed parameters of the Jansen-Rit neural mass model.

Parameter name	Value	Description
A	3.25	EPSP—Average synaptic gain
B	22.0	IPSP—Average synaptic gain
a	0.1	The average synaptic time constant
b	0.05	The average synaptic time constant
v0	6.0	Firing threshold (PSP)
ν_max_	0.0025	Maximum firing rate of the neural population
r	0.56	Steepness of the sigmoidal transformation
J	135.0	Average number of synapses between populations
α_1_	1.0	Average probability of synaptic contacts in the feedback excitatory loop
α_2_	0.8	Average probability of synaptic contacts in the slow feedback excitatory loop
α_3_	0.25	Average probability of synaptic contacts in the feedback inhibitory loop
α_4_	0.25	Average probability of synaptic contacts in the slow feedback inhibitory loop
mu	0.22	Mean input firing rate

These 19 ROIs, each modelled by Jansen-Rit model defined in (Eq. 2), were coupled through a realistic connectivity matrix which was scaled by a coupling scale factor "c". To achieve accurate predictions of neural dynamics and neurostimulation responses, we also considered variability in connection delays, which has been demonstrated to bring simulated network dynamics closer to the real neural data^49–51^. When coupled together, regional oscillator activities are dynamically modulated through the interactions with the rest of network and inputs from other regions, so that collective behavior of interacting neural mass models reproduces key features of brain dynamics. Moreover, during the simulation an additive noise with a standard deviation of 1e-6 was numerically integrated by applying the Heun stochastic algorithm, as implemented in The Virtual Brain (TVB) software^52^.

The connectivity matrix was obtained from applying functional connectivity analysis to the concurrently collected fMRI data. We used Statistical Parameter Mapping (SPM 12) toolbox to extract representative BOLD time-series from these 19 ROIs using volume of interest (VOI) analysis implemented in SPM. We focused on the t-statistic maps obtained from conflict processing (with the correct incongruent versus correct congruent contrast), and used voxels with a more liberal statistical significance of *p* < 0.05 uncorrected and created an outer sphere with the radius of 10 mm at the MNI coordinates of the local maxima of previously detected clusters. This outer sphere constrained the movement of the smaller inner sphere, with the radius of 5 mm, to the global maximum for each individual. Accordingly, we extracted the representative time-series for each region as the first principal component of the confound-corrected time-series in each selected ROI. Next, Fisher's z-transformation of the Pearson correlation coefficients of the extracted BOLD time-series was used to estimate the inter-regional connectivity measures. The connectivity matrix was then normalized so that the maximum absolute value of the cumulative input to any region, Fig. 4d. Notably the connectivity matrix was achieved independently from EEG recording. Moreover, a conduction speed of 3 (mm/ms), as proposed by Sanz Leon et al.^52^, was considered for these connections, and the delays were calculated based on the assumed conduction speed and the Euclidean distance between each pair of ROIs, Fig. 4e.

The coupling parameter "c" was multiplied by the connectivity matrix to scaling it and served as a tuning parameter that used to fit the model to responses (i.e., Hit vs. Error). We optimized the coupling parameter "c" by feeding the set of signals simulated by the Jansen-Rit neural mass model into the trained CNN. The search range for the global coupling parameter "c" spanned from 0.0001 to 0.05, with steps of 0.0002. Therefore, the search of "c" was essentially targeted at minimizing the loss of CNN which was a binary cross-entropy loss. We hypothesized that the optimal working point of the Jansen-Rit neural mass model was achieved when the CNN achieved the Hit score significantly above the chance level determined by the 5000-resampling bootstrap test, Fig. 4f.

### Statistical analysis

For the EEG-fMRI data, the statistical test on the fMRI data was not part of the hypothesis testing, rather it was for mere localization of the core cognitive network. Therefore, we used a liberal statistical significance threshold of uncorrected *p* < 0.001. Moreover, we only included clusters with the minimum extent of 10 voxels and the local maxima within the cerebral cortex (i.e., excluded clusters in white matter or cerebellum). For any other statistical inference, like determining the significance of MAE, and estimating the accuracies for the optimization of coupling scale factor or the effect of in silico stimulation, we employed non-parametric bootstrap tests with 5000 resamplings. To determine the level of significance, we compared the observed t-statistic from the original data with the bootstrapped distribution. The *p*-value was calculated by dividing the count of iterations where the resampled statistics exceeded the original statistics by the total number of iterations.

For the psychophys-fMRI data, we performed a separate GLM for every voxel where the task design was modelled with a box-car signal convolved with the hemodynamic response to isolate the effect during the condition of interest (i.e., FEEL). Voxel-level hypotheses were tested using multivariate parametric statistics with random effects across subjects and sample covariance estimation across multiple measurements. Inferences were performed at the cluster-level using Gaussian Random Field theory. The obtained results were subjected to thresholding, employing a voxel-level statistical significance threshold of *p* < 0.001, and further applying FDR-correction for the cluster level.

### Supplementary Information

Supplementary Information.

## Data Availability

We used two open access datasets that are already shared on a public repository. The links to the data have been provided in the manuscript.

## Code availability

The code for reproducing results for Figs. 3 and 4 are shared on an open access repository at https://github.com/Behzad-Iravani/CognitiveControl.git.
