# Supplementary material for: Preparatory activity of anterior insula predicts conflict errors: integrating convolutional neural networks and neural mass models
Source: Sci Rep. 2024 Jul 19;14:16682. doi: 10.1038/s41598-024-67034-5 (PMC11271609; doi:10.1038/s41598-024-67034-5)
Supplement: Supplementary file 1 — Supplementary Information. [file 41598_2024_67034_MOESM1_ESM.docx]

Preparatory Activity of Anterior Insula Predicts Conflict Errors: Integrating Convolutional Neural Networks and Neural Mass Models

Neda Kaboodvand, Hanie Karimi, and Behzad Iravani

# Supplementary figures:


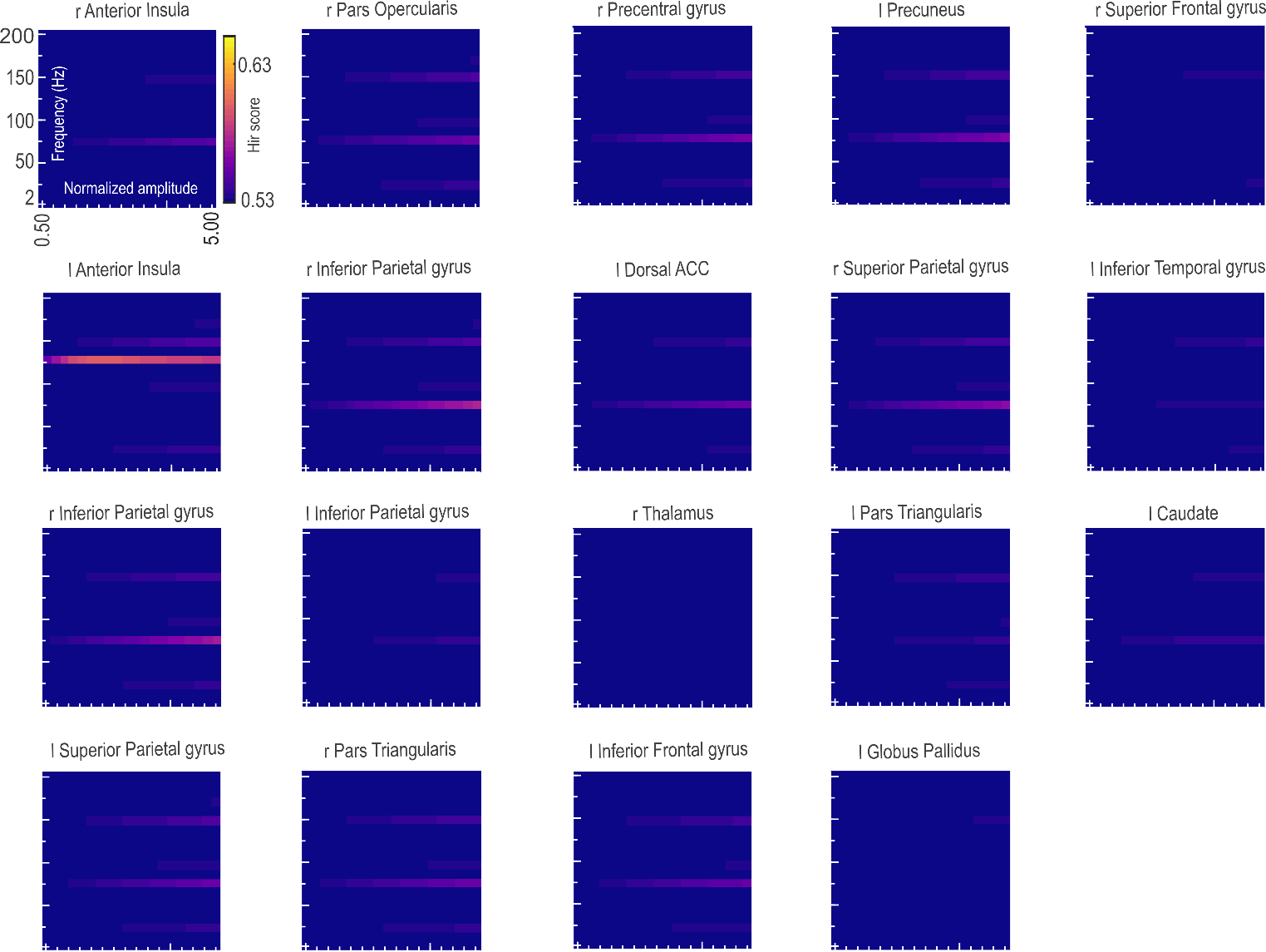


**Figure S1. Stimulation of left anterior insula at about 130 Hz increased the Hit score.** The heat maps depict the hit score as a function of in-silico stimulation amplitude and frequency. The horizontal axis represents the normalized amplitude, while the vertical axis represents the stimulation frequency. The hit score is color-coded, and the color-bar on the first panel's right side displays the associated values.

**
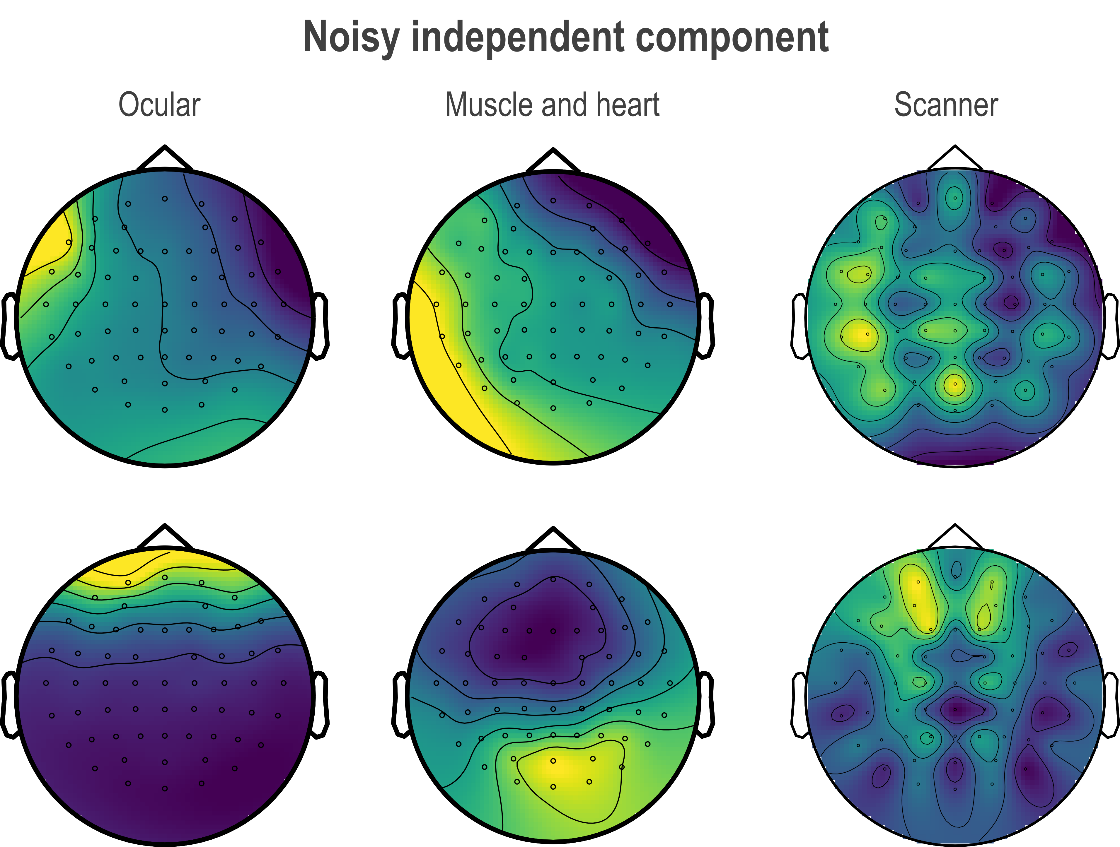
**

**Figure S2. Representative independent components that were removed for denoising EEG.** Three main categories of artifactual patterns were identified and eliminated from the EEG signals before source reconstruction, including ocular, muscle and heart, and scanner patterns.

# Supplementary tables:

**Table S1. The demographic information of the first dataset: EEG- fMRI dataset.**

| ID | Age | Sex |
| --- | --- | --- |
| 1 | 20 | F |
| 2 | 20 | M |
| 3 | 21 | M |
| 4 | 20 | M |
| 5 | 20 | F |
| 6 | 20 | F |
| 7 | 21 | M |
| 8 | 22 | M |
| 9 | 20 | M |
| 10 | 20 | M |
| 11 | 23 | F |
| 12 | 21 | F |
| 13 | 25 | M |
| 14 | 22 | F |
| 15 | 21 | F |
| 16 | 21 | F |
| 17 | 24 | M |
| 18 | 25 | F |
| 19 | 25 | F |
| 20 | 21 | F |
| 21 | 19 | M |
| 22 | 19 | F |
| 23 | 22 | F |
| 24 | 24 | F |
| 25 | 22 | F |
| 26 | 22 | F |
| 27 | 20 | F |
| 28 | 27 | M |
| 29 | 26 | M |
| 30 | 20 | M |
| 31 | 24 | M |
| 32 | 20 | F |
| 33 | 24 | F |
| 34 | 32 | M |
| 35 | 21 | F |
| 36 | 20 | F |
| 37 | 22 | F |

**Table S2. The demographic information of the second dataset: psychophys-fMRI dataset.**

| ID | Age | Sex |
| --- | --- | --- |
| sub-001 | 35 | M |
| sub-003 | 30 | F |
| sub-004 | 50 | F |
| sub-008 | 28 | F |
| sub-009 | 61 | F |
| sub-010 | 54 | F |
| sub-012 | 64 | M |
| sub-013 | 25 | M |
| sub-014 | 20 | F |
| sub-018 | 35 | M |
| sub-021 | 60 | F |
| sub-022 | 27 | F |
| sub-023 | 28 | F |
| sub-024 | 20 | M |
| sub-028 | 25 | F |
| sub-035 | 53 | F |
| sub-036 | 46 | M |
| sub-039 | 22 | F |
| sub-040 | 56 | F |
| sub-042 | 33 | F |
| sub-043 | 36 | M |
| sub-044 | 26 | M |
| sub-045 | 54 | M |
| sub-047 | 47 | F |
| sub-050 | 26 | F |
| sub-055 | 33 | F |
| sub-056 | 20 | M |
| sub-057 | 56 | F |
| sub-058 | 36 | F |
| sub-060 | 58 | M |
| sub-062 | 46 | F |
| sub-063 | 63 | F |
| sub-064 | 53 | F |
| sub-066 | 55 | M |
| sub-067 | 35 | F |
| sub-068 | 41 | F |
| sub-069 | 33 | M |
| sub-070 | 35 | F |
| sub-072 | 27 | M |
| sub-073 | 40 | F |
| sub-074 | 44 | F |
| sub-075 | 40 | F |
| sub-076 | 32 | F |
| sub-077 | 33 | M |
| sub-078 | 28 | M |
| sub-080 | 42 | F |
| sub-081 | 34 | F |
| sub-083 | 48 | M |
| sub-084 | 27 | F |
| sub-087 | 29 | F |
| sub-088 | 50 | F |
| sub-089 | 27 | F |
| sub-090 | 55 | F |
| sub-091 | 59 | F |
| sub-093 | 24 | M |
| sub-097 | 41 | F |
| sub-098 | 18 | M |
| sub-100 | 23 | F |
| sub-103 | 64 | F |
| sub-104 | 23 | F |
| sub-105 | 34 | F |
| sub-106 | 54 | F |
| sub-107 | 62 | M |
| sub-108 | 20 | F |
| sub-111 | 50 | F |
| sub-113 | 24 | F |
| sub-114 | 22 | F |

**Table S3. List of clusters related to the emotional valence volition.** For all significant clusters (size p-FDR < .05), the MNI coordinates as well as the voxel and cluster level statistics are shown.

| x | y | z | size | p-FEW size | p-FDR size | p-unc peak | p-FEW peak | p-unc |
| --- | --- | --- | --- | --- | --- | --- | --- | --- |
| -16 | -68 | +04 | 5348 | 0.000000 | 0.000000 | 0.000000 | 0.010708 | 0.000000 |
| -42 | -24 | +14 | 2924 | 0.000000 | 0.000000 | 0.000000 | 0.014002 | 0.000000 |
| +58 | -14 | +24 | 2014 | 0.000000 | 0.000000 | 0.000000 | 0.047332 | 0.000001 |
| +02 | +14 | +36 | 1947 | 0.000000 | 0.000000 | 0.000000 | 0.200405 | 0.000003 |
| +38 | +20 | -02 | 1668 | 0.000000 | 0.000000 | 0.000000 | 0.033048 | 0.000000 |
| -12 | -44 | +64 | 215 | 0.005750 | 0.003387 | 0.000261 | 0.519740 | 0.000013 |
| -54 | -70 | +02 | 190 | 0.011017 | 0.005287 | 0.000500 | 0.749554 | 0.000029 |
| +10 | -14 | +06 | 187 | 0.011932 | 0.005287 | 0.000542 | 0.572914 | 0.000016 |
| -62 | -22 | +38 | 157 | 0.027084 | 0.010750 | 0.001240 | 0.349383 | 0.000007 |
| +50 | -62 | -02 | 150 | 0.032994 | 0.011823 | 0.001516 | 0.852535 | 0.000043 |
| +14 | +10 | +00 | 127 | 0.064195 | 0.021254 | 0.002997 | 0.705293 | 0.000025 |
| +18 | -70 | -36 | 100 | 0.144556 | 0.045848 | 0.007054 | 0.636485 | 0.000020 |
| -46 | +46 | +18 | 97 | 0.158428 | 0.046753 | 0.007792 | 0.920258 | 0.000061 |

p-unc: uncorrected p-value

**Table S4. List of clusters related to the emotional arousal volition**. For all significant clusters (size p-FDR < .05), the MNI coordinates as well as the voxel and cluster level statistics are shown.

| x | y | z | size | p-FEW size | p-FDR size | p-unc peak | p-FEW peak | p-unc |
| --- | --- | --- | --- | --- | --- | --- | --- | --- |
| 32 | 16 | -8 | 1822 | 0 | 0 | 0 | 0.006848 | 0 |
| 20 | -26 | 66 | 320 | 0.000651 | 0.000681 | 0.000031 | 0.665343 | 0.000023 |
| -52 | -2 | 18 | 130 | 0.068305 | 0.049296 | 0.003361 | 0.971413 | 0.000099 |

p-unc: uncorrected p-value
